# Clinical significance of radiological pleuroparenchymal fibroelastosis pattern in interstitial lung disease patients registered for lung transplantation: a retrospective cohort study

**DOI:** 10.1186/s12931-018-0860-6

**Published:** 2018-08-30

**Authors:** Kiminobu Tanizawa, Tomohiro Handa, Takeshi Kubo, Toyofumi F. Chen-Yoshikawa, Akihiro Aoyama, Hideki Motoyama, Kyoko Hijiya, Akihiko Yoshizawa, Yohei Oshima, Kohei Ikezoe, Shinsaku Tokuda, Yoshinari Nakatsuka, Yuko Murase, Sonoko Nagai, Shigeo Muro, Toru Oga, Kazuo Chin, Toyohiro Hirai, Hiroshi Date

**Affiliations:** 1 Department of Respiratory Care and Sleep Control Medicine, Graduate School of Medicine, Kyoto University, 54 Shogoin Kawaharacho, Sakyo-ku, Kyoto 606-8507 Japan; 2 Department of Respiratory Medicine, Graduate School of Medicine, Kyoto University, 54 Shogoin Kawaharacho, Sakyo-ku, Kyoto 606-8507 Japan; 3 Department of Diagnostic Imaging and Nuclear Medicine, Graduate School of Medicine, Kyoto University, 54 Shogoin Kawaharacho, Sakyo-ku, Kyoto 606-8507 Japan; 4 Department of Thoracic Surgery, Graduate School of Medicine, Kyoto University, 54 Shogoin Kawaharacho, Sakyo-ku, Kyoto 606-8507 Japan; 5 Department of Diagnostic Pathology, Graduate School of Medicine, Kyoto University, 54 Shogoin Kawaharacho, Sakyo-ku, Kyoto 606-8507 Japan; 6 Department of Rehabilitation, Kyoto University Hospital, 54 Shogoin Kawaharacho, Sakyo-ku, Kyoto 606-8507 Japan; 7 Kyoto Central Clinic, Clinical Research Center, 58,56 Sanjodori Takakura Hidashihairu Masuyasho, Nakagyo-ku, Kyoto 604-8111 Japan

**Keywords:** Pleuroparenchymal fibroelastosis, Lung transplantation, Interstitial lung disease, Idiopathic pulmonary fibrosis, Survival

## Abstract

**Background:**

Radiological pleuroparenchymal fibroelastosis (PPFE) lesion is characterized by pleural thickening with associated signs of subpleural fibrosis on high-resolution computed tomography (HRCT). This study evaluated the clinical significance of radiological PPFE as an isolated finding or associated with other interstitial lung diseases (ILDs) in patients having fibrotic ILDs and registered for cadaveric lung transplantation (LT).

**Methods:**

This retrospective study included 118 fibrotic ILD patients registered for LT. Radiological PPFE on HRCT was assessed. The impact of radiological PPFE on clinical features and transplantation-censored survival were evaluated.

**Results:**

Radiological PPFE was observed in 30/118 cases (25%): definite PPFE (PPFE concentrated in the upper lobes, with involvement of lower lobes being less marked) in 12 (10%) and consistent PPFE (PPFE not concentrated in the upper lobes, or PPFE with features of coexistent disease present elsewhere) in 18 (15%). Of these, 12 had late-onset non-infectious pulmonary complications after hematopoietic stem-cell transplantation and/or chemotherapy (LONIPCs), 9 idiopathic PPFE, and 9 other fibrotic ILDs (idiopathic pulmonary fibrosis, IPF; other idiopathic interstitial pneumonias, other IIPs; connective tissue disease-associated ILD, CTD-ILD, and hypersensitivity pneumonia, HP). Radiological PPFE was associated with previous history of pneumothorax, lower body mass index, lower percentage of predicted forced vital capacity (%FVC), higher percentage of predicted diffusion capacity of carbon monoxide, less desaturation on six-minute walk test, and hypercapnia. The median survival time of all study cases was 449 days. Thirty-seven (28%) received LTs: cadaveric in 31 and living-donor lobar in six. Of 93 patients who did not receive LT, 66 (71%) died. Radiological PPFE was marginally associated with better survival after adjustment for age, sex, %FVC, and six-minute walk distance < 250 m (hazard ratio 0.51 [0.25–1.05], *p* = 0.07). After adjustment for covariates, idiopathic PPFE and LONIPC with radiological PPFE was associated with better survival than fibrotic ILDs without radiological PPFE (hazard ratio 0.38 [0.16–0.90], *p* = 0.03), and marginally better survival than other fibrotic ILDs with radiological PPFE (hazard ratio, 0.20 [0.04–1.11], *p* = 0.07).

**Conclusions:**

idiopathic PPFE and LONIPC with radiological PPFE has better survival on the wait list for LT than fibrotic ILDs without radiological PPFE, after adjustment for age, sex, %FVC, and six-minute walk distance.

**Electronic supplementary material:**

The online version of this article (10.1186/s12931-018-0860-6) contains supplementary material, which is available to authorized users.

## Background

Pleuroparenchymal fibroelastosis (PPFE) is characterised by pleural fibrosis and subpleural lung parenchymal fibroelastosis with or without upper lobe predominance [1–3]. Idiopathic PPFE has been included as a disease entity in the current guideline for classification of idiopathic interstitial pneumonias (IIPs), [4] and PPFE is also postulated as radiological and pathological patterns [2, 5].

Radiological and pathological PPFE patterns the same as those for idiopathic PPFE were reported in diseases other than idiopathic PPFE, such as late-onset non-infectious pulmonary complications after hematopoietic stem-cell transplantation and/or chemotherapy (LONIPCs) [6, 7] and restrictive chronic lung allograft dysfunction after lung transplantation (LT) [8, 9]. These patterns can also exist with other radiological and pathological patterns such as usual interstitial pneumonia (UIP) [3, 5], nonspecific interstitial pneumonia (NSIP), organizing pneumonia, and bronchiolitis obliterans [10, 11]. However, it is unknown how radiological PPFE affects the clinical features and outcomes across interstitial lung diseases (ILDs), especially in severe cases.

Of ILDs with PPFE lesions, idiopathic PPFE and LONIPC are often progressive and fatal [2, 3, 10, 12, 13], but the rate of disease progression is variable [14]. Idiopathic pulmonary fibrosis (IPF) with pathological PPFE also tends to have worse survival than IPF without pathological PPFE [5]. In idiopathic PPFE, LONIPC, and IPF, PPFE lesions are associated with restrictive impairment [1–3, 5, 15]. These findings suggest that PPFE lesions may have a significant effect on physiology and survival of ILD patients, whether the lesions are predominant (idiopathic PPFE/LONIPC) or concomitant with other predominant lesions such as IPF.

The objective of this retrospective study was to evaluate the clinical significance of radiological PPFE in patients with severe ILDs who are on a wait list for LT. We hypothesized that the presence of radiological PPFE would be associated with more severe physiological impairments and poorer survival.

## Methods

### Study population

Patients with fibrotic ILDs were identified from a prospective registry for cadaveric LT at Kyoto Universal Hospital between April 1, 2010 and August 31, 2015. Fibrotic ILDs included IPF, other IIPs, connective tissue disease-associated ILD (CTD-ILD), hypersensitivity pneumonia (HP), and LONIPCs. Registration criteria for the nationwide Japan Organ Transplant Network (JOTN) are 1) meeting the international listing criteria for LT, and 2) age < 60 years for unilateral LT, and < 55 years for bilateral LT. The algorithm for cadaveric donor lung allocation is based primarily on accrued time on the wait list, a situation which favors patients with slowly progressive diseases and disfavors patients with rapidly progressive diseases [16]. Exclusion criteria for this study were 1) age < 18 years old at the time of registration and 2) registration for a second LT. Patients with lymphangioleiomyomatosis, pulmonary, Langerhans cell histiocytosis, and sarcoidosis were excluded from analysis because these ILDs are not necessarily fibrotic and radiological PPFE did not accompany with these diseases in this cohort.

Clinical diagnoses for all patients were established at the time of evaluation for registration based on the multidisciplinary diagnoses at the individual referring institutes. For cases who had undergone LT, the final diagnoses were determined through a multidisciplinary discussion after LT at Kyoto University Hospital. idiopathic PPFE was diagnosed either by: 1) a multidisciplinary approach with pathological diagnosis of PPFE [2, 4], or 2) a radiological PPFE pattern (described below) without any alternative diagnosis.

### Radiological evaluation

**Fig. 1:**
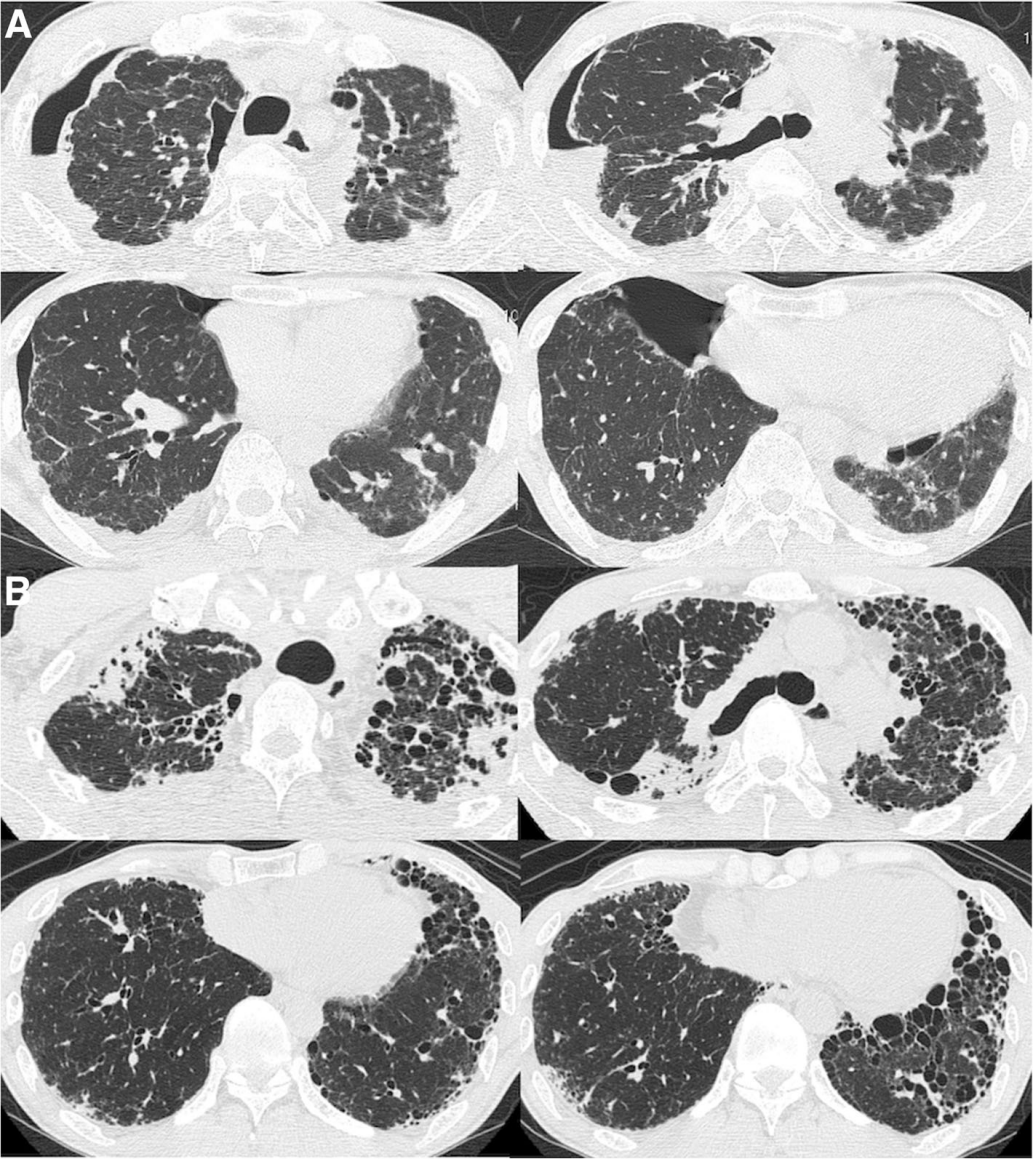
Radiological pleuroparenchymal fibroelastosis (PPFE) pattern on high-resolution computed tomography. **a**. Definite PPFE pattern. A 57-year-old male with idiopathic PPFE had definite PPFE. He received unilateral cadaveric lung transplantation after waiting for 266 days. Pathological PPFE was confirmed in the explanted lung. **b**. Consistent PPFE pattern. A 55-year-old male with IPF. Pleural thickening with associated signs of subpleural fibrosis is present in the upper lobes, while cystic lesions including honeycombing are seen in the lower lobes. Surgical lung biopsy showed pathological UIP and PPFE

Radiological PPFE patterns were defined as: *definite*, indicated by pleural thickening with associated subpleural fibrosis concentrated in the upper lobes, with involvement of lower lobes being less marked or absent (Fig. 1a), or *consistent*, indicated by upper lobe pleural thickening with associated signs of subpleural fibrosis present, but with distribution of these changes not concentrated in the upper lobes, or features of coexistent disease present elsewhere (Fig. 1b) [2]. Both definite and consistent PPFE patterns were designated as indicating radiological PPFE.

All high-resolution computed tomography scans were reviewed for this study by two observers (K.T. and T.K. who had 22 and 17 years of experience, respectively) blinded to clinical information, and the presence or absence of a radiological PPFE pattern was recorded. Inter-observer disagreements were resolved by consensus.

### Clinical variables

Baseline clinical variables prospectively obtained and included in this analysis were demographics, serum biomarkers, standardized pulmonary function tests, six-minute walk test (6MWT), and arterial blood gas analysis. Arterial blood was collected with the patient in the supine position while breathing ambient air or supplementary oxygen as if at rest. The percutaneous oxygen saturation (SpO_2_) level was monitored continuously during the 6MWT.

### Statistical analysis

Clinical features were summarized using medians (interquartile range [IQR]) and numbers (percentage) as appropriate. Wilcoxon and Fisher’s exact tests were used for group comparisons. Survival time was calculated from the registration date to the JOTN until the patient’s death, with patients right-censored at the time of cadaveric or living-donor lobar LT, or the last contact. The study population was divided into three categories defined by radiological PPFE and diagnoses: 1) idiopathic PPFE and LONIPC with radiological PPFE (all patients had radiological PPFE) (idiopathic PPFE/LONIPC), 2) other fibrotic ILDs (IPF, other IIPs, CTD-ILD, and HP) with radiological PPFE, and 3) fibrotic ILDs without radiological PPFE. For some analyses, the first category was further subdivided into idiopathic PPFE and LONIPC with radiological PPFE, making four categories. Kaplan-Meier curves and the log-rank test were used to demonstrate and compare overall survival time by radiological PPFE and the cohort divided into either three or four categories. Cox regression was performed to adjust survival analysis for age, sex, and the percentage of predicted forced vital capacity (%FVC). These variables were identified as potential confounders between radiological PPFE and survival, and between either three or four categories and survival. The percentage of predicted diffusion capacity of carbon monoxide (%DL_CO_) was not included in multivariable models because %DL_CO_ was available only in 74 patients (63%). Among data gained from the 6MWT, six-minute walk distance (6MWD) < 250 m was included as a variable because it has been adopted in the listing criteria for LT [17], although the 6MWT has not been standardized for the usage of oxygen when comparing different patients [18]. Competing risk analysis establishing LT as a competitive outcome was also performed. All data analyses were performed using STATA/IC 14.2 for Mac (Stata Corp., Lakeway, TX, USA), with statistical significance set at *p* < 0.05.

## Results

### Patients’ characteristics

One hundred eighteen patients with ILDs were identified from the Kyoto University Hospital registry for cadaveric LT. Radiological PPFE was diagnosed in 30/118 (25%) patients, with 12 (10%) diagnosed with definite and 18 (15%) diagnosed with consistent PPFE. All cases with radiological PPFE had traction bronchiectasis or volume loss of upper lobes, suggesting some clinical significance beyond the apical cap. Of these, 12 had LONIPC, with idiopathic PPFE (*n* = 9) as the second most common diagnosis (Table 1). Of the 12 patients with definite PPFE, six had LONIPC, five had idiopathic PPFE, and one had CTD-ILD. Pathological diagnosis was achieved in 78/118 (66%) cases. Sixteen of the 30 cases with radiological PPFE (53%) had a pathological diagnosis, with histopathological PPFE confirmed in 12 (40%) of the radiological PPFE cases. In these 12 cases, histopathology showed predominant PPFE in four with idiopathic PPFE and one with LONIPC, coexistent PPFE and bronchiolitis in four with LONIPC; predominant UIP with coexistent PPFE in two with IPF, and predominant non-specific interstitial pneumonia with coexistent PPFE in one with LONIPC (Additional file 1: Table S1). Four cases with radiological PPFE (two with other IIPs, one with IPF and one with CTD-ILD) did not have histopathological PPFE although a pathological diagnosis was available. None of these four cases underwent LT and only surgical lung biopsy samples were available for diagnosis (Additional file 1: Table S1). No case without radiological PPFE had histopathological PPFE. Baseline clinical characteristics are compared between patients with and without radiological PPFE in Table 2. Radiological PPFE was associated with female gender, never-smoking, previous history of pneumothorax, lower body mass index (BMI), lower levels of serum Krebs von der Lungen-6 and lactate dehydrogenase, lower %FVC, higher %DL_CO_, less desaturation on the 6MWT, and hypercapnia.

**Table 1:** Diagnoses of study population (*n* = 118)

	With radiological PPFE			Without radiological PPFE
	Total	Definite	Consistent	
Number	30 (25.4)	12 (10.2)	18 (15.3)	88 (74.6)
LONIPC	12 (40.0)	6 (50.0)	6 (33.3)	2 (2.3)
Idiopathic PPFE	9 (30.0)	5 (41.7)	4 (22.2)	0 (0.0)
IPF	4 (13.3)	0 (0.0)	4 (22.2)	33 (37.5)
Other IIPs	2 (6.7)	0 (0.0)	2 (11.1)	21 (23.9)
CTD-ILD	2 (6.7)	1 (8.3)	1 (5.6)	25 (28.4)
HP	1 (3.3)	0 (0.0)	1 (5.6)	7 (8.0)

Data are presented as number (percentage)

Abbreviations: *PPFE* plueroparenchymal fibroelastosis, *LONIPC* late-onset non-infectious pulmonary complication after hematopoetic stem-cell transplantation and/or chemotherapy, *IPF* idiopathic pulmonary fibrosis, *other IIPs* other idiopathic interstitial pneumonias than idiopathic pulmonary fibrosis, *CTD-ILD* connective tissue disease-associated interstitial lung disease, *HP* hypersensitivity pneumonia

**Table 2:** Demographics, serum biomarkers, pulmonary function tests, six-minute walk test, arterial blood gas analysis, and treatment at the time of registration for lung transplantation (*n* = 118)

	With rPPFE	Without rPPFE	*P*
Number	30	88	
Demographics			
Age	45.5 (37, 51)	51 (43, 56)	0.05
Male	13 (43.3)	58 (65.9)	0.03
Ever-smoking	8 (26.7)	54 (61.4)	0.001
Previous pneumothorax	24 (80.0)	23 (26.1)	< 0.001
Family history	2 (6.7)	21 (23.9)	0.16
mMRC [1]	3 (3, 4)	4 (3, 4)	0.15
BMI	15.9 (14.8, 17.2)	21.5 (18.8, 25.1)	< 0.001
Serum biomarkers			
KL-6, IU/L	542 (402, 698)	1460 (933, 2150)	< 0.001
LDH, IU/L	186.5 (172, 212)	229 (202, 271)	< 0.001
Pulmonary function tests			
%FVC	35.75 (22.6, 46.2)	48.5 (37.6, 62.0)	< 0.001
%DLCO	45.5 (37, 51)	24.4 (17.4, 31.9)	0.002
Six-minute walk test			
Distance, meter	375 (243, 500)	333 (235, 454)	0.36
Distance < 250 m	8 (26.7)	26 (29.5)	0.82
Lowest SpO_2_, %	87.5 (81, 93)	81 (76, 87)	< 0.001
Arterial blood gas analysis			
PaO_2_, Torr	77.8 (65.4, 86.6)	74.05 (63.4, 87.9)	0.73
PaCO_2_, Torr	49.55 (45.6, 56.9)	43.55 (40.7, 47.55)	< 0.001
Treatment			
Corticosteroid	11 (36.7)	62 (70.5)	0.002
IS agent	3(10.0)	40 (45.5)	< 0.001
Antifibrotic agent	7 (23.3)	39 (44.3)	0.05
LTOT	15 (50.0)	63 (71.6)	0.04

Data are presented as number (percentage) or median (interquartile range)

Abbreviations: *PPFE* plueroparenchymal fibroelastosis, *rPPFE* radiological PPFE, *mMRC* modified Medical Research Council dyspnea scale, *BMI* body mass index, *KL*-6 Krebs von der Lungen-6, *LDH* lactate dehydrogenase, %*FVC* the percentage to predicted forced vital capacity, %*DLCO* the percentage to predicted diffusion capacity of carbon monoxide, *SpO*_2_ percutaneous oxygen saturation, *PaO*_2_ arterial partial pressure of oxygen, *PaCO*_2_ arterial partial pressure of carbon dioxide, *IS* immunosuppressive, *LTOT* long-term oxygen therapy

### Radiological PPFE association with survival

The median survival time of the 118 patients was 449 days (IQR: 215, 749). Thirty-five (30%) received LTs: cadaveric in 29 (unilateral in 24, and bilateral in five) and living-donor lobar in six. Of 83 patients who did not receive LT, 63 (76%) died. The causes of death were chronic respiratory failure in 35 (56%), acute exacerbation of ILDs in 15 (24%), and pneumonia/infection in three (5%).

**Fig. 2:**
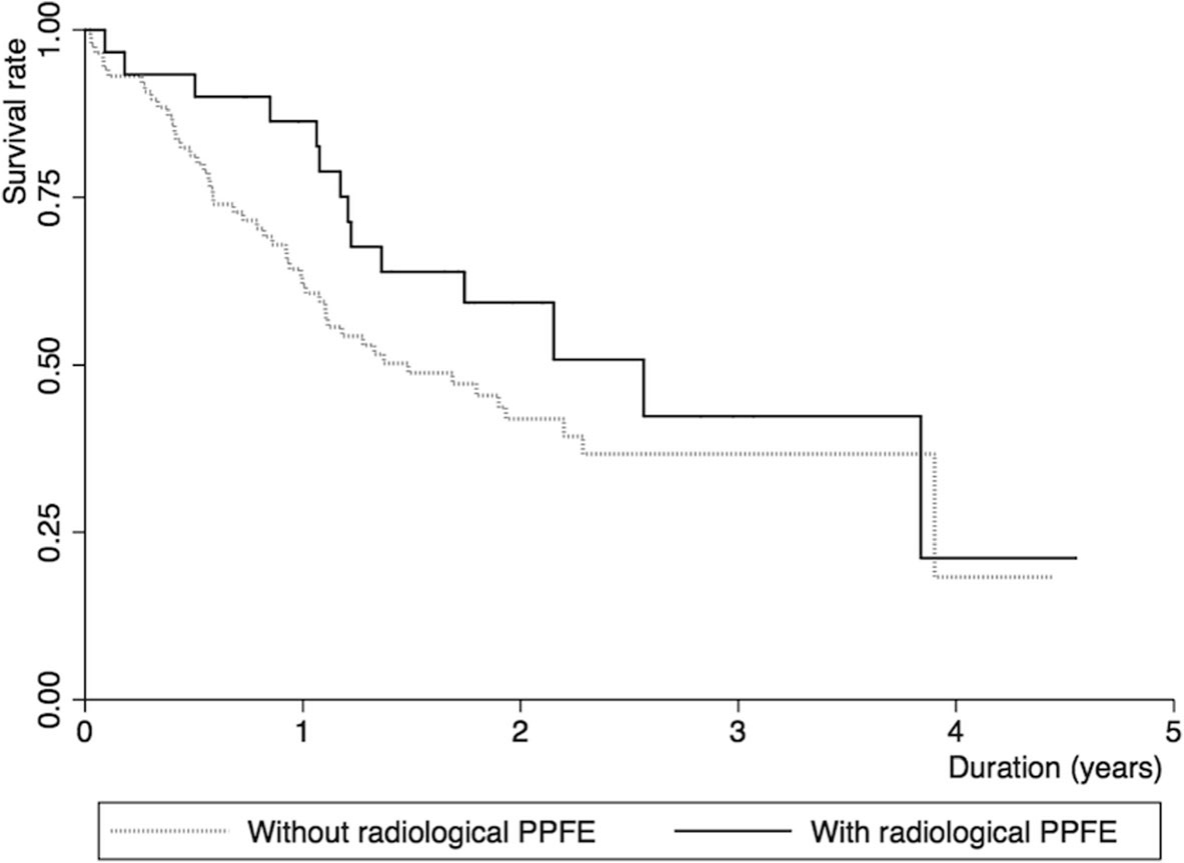
Kaplan-Meier survival estimates. Solid and dotted lines represent patients with and without radiological pleuroparenchymal fibroelastosis (PPFE) (*n* = 30; *n* = 88), respectively

Kaplan-Meier survival estimates are shown for patients with and without PPFE (Fig. 2a). The log-rank *p* value was not significant (*p* = 0.17). The Cox regression model adjusted for age, sex, %FVC, and 6MWD < 250 m showed a marginal association of radiological PPFE with better survival (hazard ratio [HR], 0.51; 95% confidence interval [95%CI], 0.25–1.05, *p* = 0.07) (Table 3, *Model 1*). Competing risk analysis showed similar results (Table 3, *Model 1*).

**Table 3:** Multivariable Cox proportional hazards model for mortality (*n* = 118)

	*Transplant-censored*			Competing analysis		
*Model 1*	Hazard ratio	95%CI	*p* value	Hazard ratio	95%CI	*P* value
Radiological PPFE	0.51	(0.25, 1.05)	0.07	0.57	(0.30, 1.12)	0.10
Age	1.04	(1.01, 1.07)	0.01	1.03	(1.01, 1.06)	0.02
Male	1.05	(0.61, 1.82)	0.86	0.88	(0.51, 1.52)	0.65
%FVC	0.97	(0.95, 1.00)	0.03	0.98	(0.96, 1.00)	0.07
Six-minute walk distance < 250 m	1.87	(1.02, 3.43)	0.04	2.36	(1.33, 4.19)	0.003
*Model 2*						
Category						
Fibrotic ILDs^a^ without radiological PPFE	Ref.	–	–	Ref.	–	–
Idiopathic PPFE and LONIPC with radiological PPFE	0.38	(0.16, 0.90)	0.03	0.41	(0.21, 0.80)	0.01
Other fibrotic ILDs with radiological PPFE	0.96	(0.35, 2.64)	0.94	1.19	(0.51, 2.78)	0.69
Age	1.03	(1.00, 1.07)	0.02	1.03	(1.00, 1.06)	0.04
Male	1.01	(0.58, 1.75)	0.98	0.85	(0.49, 1.47)	0.56
%FVC	0.98	(0.95, 1.00)	0.03	0.98	(0.97, 1.00)	0.06
Six-minute walk distance < 250 m	1.99	(1.07, 3.70)	0.03	2.51	(1.39, 4.54)	0.002
*Model 3*						
Category						
Fibrotic ILDs without radiological PPFE	Ref.	–	–	Ref.	–	–
Idiopathic PPFE	0.35	(0.12, 1.04)	0.06	0.39	(0.20, 0.78)	0.01
LONIPC with radiological PPFE	0.42	(0.12, 1.50)	0.18	0.43	(0.35, 2.64)	0.15
Other fibrotic ILDs^a^ with radiological PPFE	0.96	(0.35, 2.64)	0.94	1.19	(0.51, 2.78)	0.69
Age	1.04	(1.00, 1.07)	0.02	1.03	(1.00, 1.07)	0.04
Male	1.01	(0.58, 1.75)	0.98	0.85	(0.58, 1.75)	0.57
%FVC	0.98	(0.95, 1.00)	0.03	0.98	(0.95, 1.00)	0.06
Six-minute walk distance < 250 m	2.00	(1.07, 3.74)	0.03	2.52	(1.39, 4.54)	0.003

^a^Other fibrotic ILDs are IPF, other IIPs, CTD-ILD, and HP

Abbreviations: *ILD* interstitial lung disease, *PPFE* plueroparenchymal fibroelastosis, *LONIPC* late-onset non-infectious pulmonary complication after hematopoetic stem-cell transplantation and/or chemotherapy, %*FVC* the percentage to predicted forced vital capacity, *IPF* idiopathic pulmonary fibrosis, *other IIPs* other idiopathic interstitial pneumonias than idiopathic pulmonary fibrosis, *CTD-ILD* connective tissue disease-associated interstitial lung disease, *HP* hypersensitivity pneumonia

**Fig. 3:**
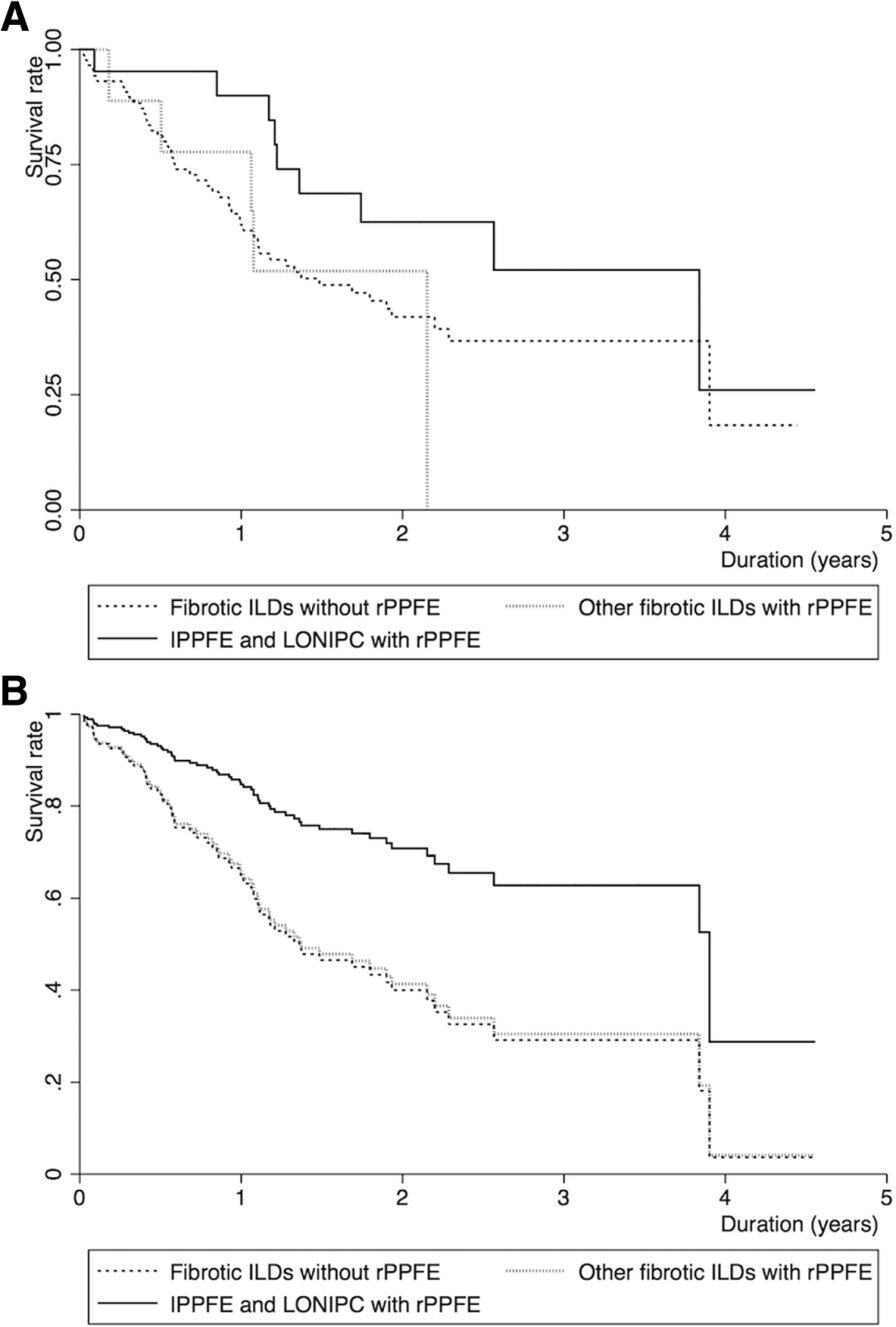
Kaplan-Meier survival estimates and estimated survival curves. **a**. Kaplan-Meier survival estimates. Solid, dotted, and short dashed lines represent idiopathic PPFE and LONIPC with radiological PPFE, other fibrotic ILDs (IPF, other IIPs, CTD-ILD, and HP) with radiological PPFE, and fibrotic ILDs without radiological PPFE (*n* = 21; *n* = 9; *n* = 88; *n* = 13), respectively. **b**. Estimated Cox survival curves adjusted for age, sex, and %FVC. Solid, dotted, and short dashed lines represent idiopathic PPFE and LONIPC with radiological PPFE, other fibrotic ILDs (IPF, other IIPs, CTD-ILD, and HP) with radiological PPFE, and fibrotic ILDs without radiological PPFE, respectively. Abbreviations: idiopathic PPFE, idiopathic pleuroparenchymal fibroelastosis; LONIPC, late-onset non-infectious pulmonary complication after hematopoetic stem-cell transplantation and/or chemotherapy; rPPFE, radiological PPFE; ILD, interstitial lung disease; IPF, idiopathic pulmonary fibrosis; IIPs, idiopathic interstitial pneumonias; CTD-ILD, connective tissue disease-associated ILD; HP, hypersensitivity pneumonia

Kaplan-Meier survival estimates are shown in Fig. 3a for cases divided into three categories by radiological PPFE and etiologies. The log-rank *p* value was not significant for idiopathic PPFE/LONIPC with radiological PPFE compared with fibrotic ILDs without radiological PPFE (the reference) (*p* = 0.10), or for other fibrotic ILDs with radiological PPFE compared to the reference (*p* = 0.99). However, the Cox regression model adjusted for age, sex, %FVC, and 6MWD < 250 m showed an association of idiopathic PPFE/LONIPC with better survival (HR, 0.38; 95%CI, 0.16–0.90, *p* = 0.03; competing analysis: HR, 0.41; 95%CI, 0.21–0.80, *p* = 0.01) (Table 3, *Model 2* and Fig. 3b). Survival for idiopathic PPFE/LONIPC with radiological PPFE was similar to that for other fibrotic ILDs with radiological PPFE (log-rank, *p* = 0.14). After adjustment for age, sex, %FVC, and 6MWD < 250 m, idiopathic PPFE/LONIPC with radiological PPFE was marginally/significantly associated with better survival in comparison with other fibrotic ILDs with radiological PPFE (HR, 0.20; 95%CI, 0.04–1.11, *p* = 0.07; competing analysis: HR, 0.17; 95%CI, 0.04–0.76, *p* = 0.02).

**Fig. 4:**
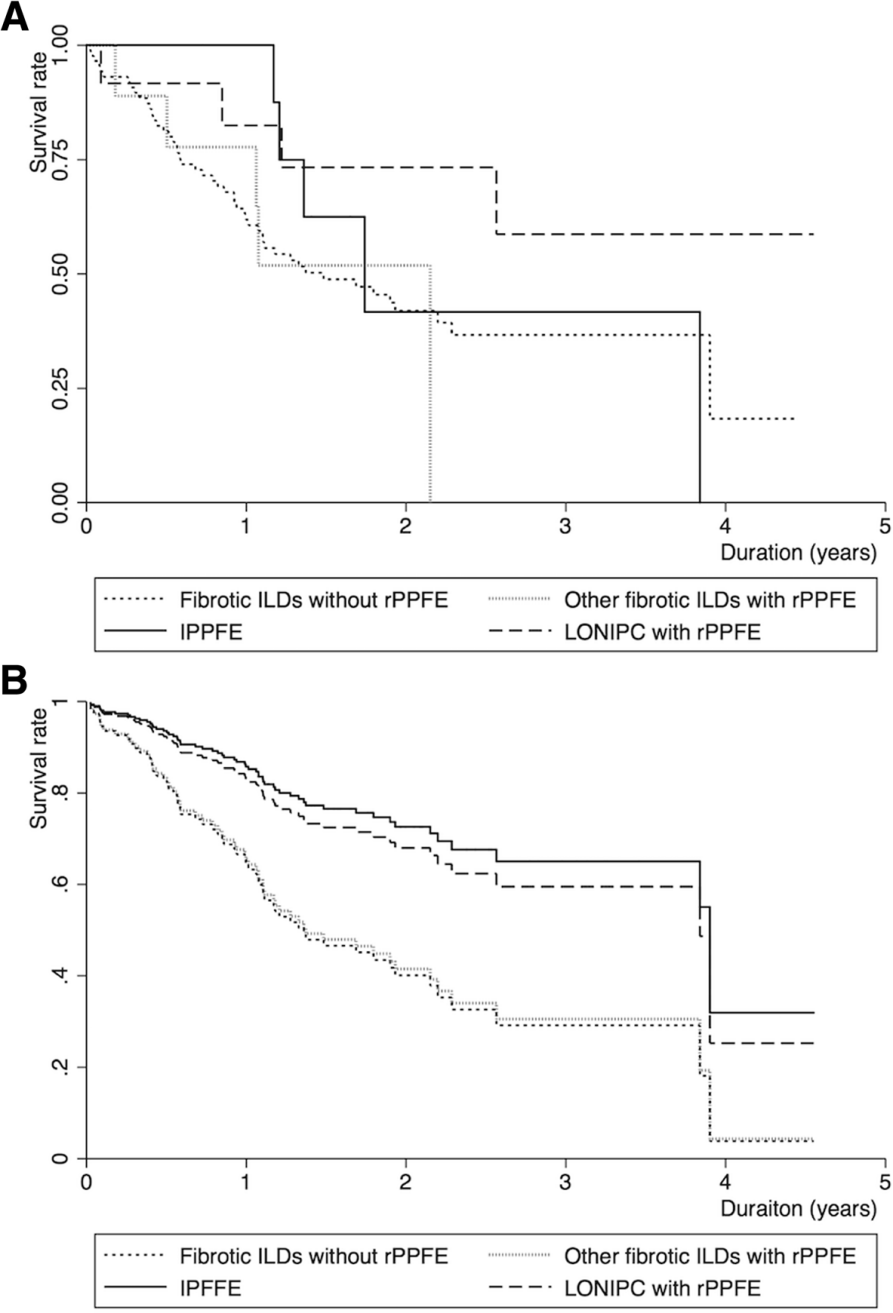
Kaplan-Meier survival estimates and estimated survival curves. **a**. Kaplan-Meier survival estimates. Solid, dotted, short dashed, and long dashed lines represent idiopathic PPFE, other fibrotic ILDs (IPF, other IIPs, CTD-ILD, and HP) with radiological PPFE, fibrotic ILDs without radiological PPFE, and LONIPC with radiological PPFE (n = 9; n = 9; n = 88; *n* = 12), respectively. **b**. Estimated Cox survival curves adjusted for age, sex, and %FVC. Solid, dotted, short dashed, and long dashed lines represent idiopathic PPFE, other fibrotic ILDs (IPF, other IIPs, CTD-ILD, and HP) with radiological PPFE, fibrotic ILDs without radiological PPFE, and LONIPC with radiological PPFE, respectively

Figure 4a shows Kaplan-Meier survival estimates after dividing idiopathic PPFE/LONIPC with radiological PPFE into two subgroups: idiopathic PPFE and LONIPC with radiological PPFE. The log-rank *p* value was not significant for idiopathic PPFE compared to fibrotic ILDs without radiological PPFE (the reference) (*p* = 0.61), or for LONIPC with radiological PPFE compared to the reference (*p* = 0.09). The Cox regression model adjusted for age, sex, %FVC, and 6MWD < 250 m showed marginal/significant associations of idiopathic PPFE with better survival (HR, 0.35; 95%CI, 0.12–1.04, *p* = 0.06; competing analysis: HR, 0.39; 95%CI, 0.20–0.78, *p* = 0.01) (Table 3, *Model 3*, and Fig. 4b).

## Discussion

In our cohort of fibrotic ILD patients registered for LT, 25% had radiological PPFE: the most common diagnosis in those with radiological PPFE was LONIPC, followed by idiopathic PPFE and IPF. Radiological PPFE was associated with a previous history of pneumothorax, lower BMI, lower %FVC, higher %DL_CO_, less desaturation on the 6MWT, and hypercapnia. Idiopathic PPFE/LONIPC with radiological PPFE was significantly associated with better survival than fibrotic ILDs without radiological PPFE, after adjustment for demographics, %FVC, and 6MWD. However, further studies are needed to confirm these findings and revise the registration criteria.

The impact of radiological PPFE on survival was different between idiopathic PPFE/LONIPC and other fibrotic ILDs. In idiopathic PPFE/LONIPC, PPFE lesions are predominant, and these lesions worsen at variable rates of progression [14]. In other fibrotic ILDs with radiological PPFE, other lesions than PPFE, such as UIP and NSIP, are predominant, and they may influence survival independently from PPFE. Our results suggest that diagnosis of idiopathic PPFE may affect survival more strongly than the presence of radiological PPFE.

Reported survival time of patients with coexistent PPFE and other fibrotic ILDs is varied. UIP/possible UIP in the lower lobes did not affect survival of radiologically defined idiopathic PPFE patients [19]. Biopsy-proven IPF with a concomitant histopathological PPFE lesion had worse survival than biopsy-proven IPF without such a lesion [5]. In our cohort, on the other hand, patients with other fibrotic ILDs with radiological PPFE had survival time similar to those with fibrotic ILDs without radiological PPFE. This difference may be affected by the small number of cases with coexistent PPFE and other fibrotic ILDs (nine with UIP and PPFE in the biopsy-proven cohort [5], and nine with other fibrotic ILDs (IPF, other IIPs, CTD-ILD, and HP) with radiological PPFE in our cohort). Future analyses of larger cohorts may be more definitive.

Radiological PPFE was associated with several clinical and physiological characteristics. History of pneumothorax, low BMI, and low %FVC were previously reported for patients with idiopathic PPFE, LONIPC, and IPF with pathological PPFE [1, 5, 6, 15, 19]. Restrictive impairment present in patients with radiological PPFE may be partially caused by a flattened thoracic cage, in addition to lung parenchymal fibrosis. The thoracic cage is flattened and accompanied by disease progression in idiopathic PPFE [20]. Lower BMI (thin body), and hypercapnia are possibly associated with this musculoskeletal change. Lower BMI has been associated with inspiratory muscle dysfunction in cystic fibrosis [21], and inspiratory muscle training improves restrictive impairment and exercise capacity in adults with cystic fibrosis [22]. Inspiratory muscle dysfunction may also have an effect on %FVC among patients with radiological PPFE.

Higher %DL_CO_ and less desaturation on the 6MWT in patients with radiological PPFE suggest that the lung parenchyma may be relatively preserved, compared to the severity of the restrictive and ventilatory impairments. Lower levels of serum biomarkers for lung injury (Krebs von der Lungen-6 and lactate dehydrogenase) are also consistent with less damaged parenchyma [23]. The relatively milder parenchymal lesions may explain the better survival for patients with idiopathic PPFE/LONIPC, after adjustment for lower %FVC.

The diagnostic criteria for idiopathic PPFE have not been validated yet. Five of the nine patients with idiopathic PPFE in this cohort were diagnosed without the support of a pathological diagnosis, whereas the recent classification criteria for IIPs require a pathological diagnosis when diagnosing idiopathic PPFE [4]. A surgical lung biopsy for the diagnosis of idiopathic PPFE is often unavailable because of patients’ severe physiological impairment and high risks for refractory postoperative pneumothorax [12, 24]. Correlation between radiological and pathological diagnoses of PPFE seems acceptable in the literature of idiopathic PPFE [2]. Thus, we diagnosed some cases of idiopathic PPFE without pathological diagnosis, after we carefully excluded any other diagnoses. In fact, no case without radiological PPFE was shown to have histopathological PPFE in our cohort when pathological diagnosis was available.

In addition, the diagnostic criteria for radiological PPFE have been defined only descriptively [2]. Differentiation of radiological PPFE from apical cap may be difficult in some cases [12]. A recent study included radiological progression of disease in the diagnostic criteria for idiopathic PPFE to exclude apical cap [19]. All radiological PPFE cases in our cohort showed traction bronchiectasis or volume loss of the upper lobes, suggesting disease progression different from apical cap. However, more quantitative approaches may improve diagnostic accuracy and the predictive value of radiological evaluation for survival.

The present study had several limitations in terms of reduced generalizability. Our cohort excluded all patients who could not be registered for LT (age > 60 for unilateral and > 55 for bilateral LT, active infection, and other serious comorbidities). The prevalence of idiopathic PPFE/LONIPC with radiological PPFE in this cohort (18%) was higher than that of idiopathic PPFE in consecutive ILD patients undergoing SLB (5.9%) [3] and that of LONIPC with PPFE in hematopoietic stem-cell and lung transplantation recipients (0.3% in hematopoietic stem-cell recipients, and 7.5% in lung transplantation recipients) [25]. More PPFE cases may have been accumulated in our cohort of ILD patients waiting for LT than in other general ILD cohorts because there is no effective pharmacological therapy for idiopathic PPFE/LONIPC with radiological PPFE. As idiopathic PPFE/LONIPC with radiological PPFE patients are often younger than those with other fibrotic ILDs such as IPF, these patients are more likely to be referred to LT centers for registry.

Referral bias cannot be overemphasized. In particular, the average wait time for LT is longer than 800 days in Japan [16]. Some physicians may have given up referring the most severe cases to LT centers, whether or not the cases have radiological PPFE. Two phenotypes of disease progression (rapid and slow) were reported in idiopathic PPFE [14]. It is possible that our cohort mostly included patients with slowly progressive idiopathic PPFE, not another rapidly progressive phenotype, which can be associated with better survival of idiopathic PPFE and LONIPC with radiological PPFE. The algorithm for cadaveric donor lung allocation favoring patients with slowly progressive diseases may have exaggerated the trend toward better survival of this subgroup.

Although we used one of the largest cohorts of ILD patients registered for LT in Japan, only 30 patients with radiological PPFE were included. Predictors for survival of idiopathic PPFE and LONIPC and outcomes after LT should be also addressed [26, 27]. In addition, due to our retrospective design, changes in pulmonary physiology over time and treatment after registration could not be addressed.

## Conclusions

In conclusion, we demonstrated that radiological PPFE was associated with a diagnosis of idiopathic PPFE/LONIPC, previous history of pneumothorax, lower BMI, lower %FVC, higher %DL_CO_, less desaturation on the 6MWT, and hypercapnia.

Idiopathic PPFE/LONIPC with radiological PPFE was significantly associated with better survival in ILD patients registered for LT, after adjustment for its association with lower %FVC and other covariates. Among several fibrotic ILDs with radiological PPFE, idiopathic PPFE and LONIPC, especially idiopathic PPFE may be associated with better survival than fibrotic ILDs without radiological PPFE. If validated, this suggests that idiopathic PPFE should be considered differently from other ILDs when the registration criteria for LT are revised.

## Additional file

Additional file 1: **Table S1**. Sixteen radiological PPFE cases with a histopathological diagnosis. (DOCX 16 kb)

## Abbreviations

%DL_CO_: Percentage of predicted diffusion capacity of carbon monoxide
%FVC: Percentage of predicted forced vital capacity
6MWT: Six-minute walk test
95%CI: 95% confidence interval
BMI: Body mass index
BO: Bronchiolitis obliterans
CT-ILD: Connective tissue disease-associated interstitial lung disease
HP: Hypersensitivity pneumonia
HR: Hazard ratio
IIP: Idiopathic interstitial pneumonia
ILD: Interstitial lung disease
IPF: Idiopathic pulmonary fibrosis
IQR: Interquartile range
JOTN: Japan Organ Transplant Network
LAM: Lymphangioleiomyomatosis
LONIPC: Late-onset non-infectious pulmonary complication after hematopoietic stem-cell transplantation and/or chemotherapy
LT: Lung transplantation
NSIP: Nonspecific interstitial pneumonia
PLCH: Pulmonary Langerhans cell histiocytosis
PPFE: Pleuroparenchymal fibroelastosis
rPPFE: Radiological pleuroparenchymal fibroelastosis
UIP: Usual interstitial pneumonia
