# Supplementary material for: Clinical significance of radiological pleuroparenchymal fibroelastosis pattern in interstitial lung disease patients registered for lung transplantation: a retrospective cohort study
Source: Respir Res. 2018 Aug 30;19:162. doi: 10.1186/s12931-018-0860-6 (PMC6117972; doi:10.1186/s12931-018-0860-6)
Supplement: Supplementary file 1 — Table S1. Sixteen radiological PPFE cases with a histopathological diagnosis. (DOCX 16 kb) [file 12931_2018_860_MOESM1_ESM.docx]

Additional file 1: Table S1. Sixteen radiological PPFE cases with a histopathological diagnosis.

| No. | Age | Sex | Diagnosis | Radiological | Specimens | Histopathological diagnosis | | | Outcome |
| --- | --- | --- | --- | --- | --- | --- | --- | --- | --- |
|  |  |  |  | PPFE |  | PPFE | Predominant | Additional |  |
| 1 | 40 | F | Idiopathic PPFE | Consistent | SLB | + | PPFE |  | Deceased before LT |
| 2 | 51 | F | Idiopathic PPFE | Consistent | SLB | + | PPFE |  | Deceased before LT |
| 3 | 56 | M | Idiopathic PPFE | Definite | Explanted | + | PPFE |  | Deceased after cadaveric LT |
| 4 | 57 | M | Idiopathic PPFE | Definite | Explanted | + | PPFE |  | Deceased after cadaveric LT |
| 5 | 26 | M | LONIPC | Definite | Explanted | + | PPFE | BO | Deceased after cadaveric LT |
| 6 | 27 | M | LONIPC | Consistent | Explanted | + | PPFE | BO | Alive after living-donor lobar LT |
| 7 | 28 | F | LONIPC | Consistent | Explanted | + | PPFE | BO | Deceased after cadaveric LT |
| 8 | 35 | F | LONIPC | Consistent | SLB | + | NSIP | PPFE | Alive without LT |
| 9 | 39 | F | LONIPC | Definite | SLB | + | PPFE |  | Deceased before LT |
| 10 | 46 | F | LONIPC | Consistent | Explanted | + | PPFE | BO | Alive after living-donor lobar LT |
| 11 | 51 | M | IPF | Consistent | SLB | + | UIP | PPFE | Deceased before LT |
| 12 | 59 | M | IPF | Consistent | SLB | + | UIP | PPFE | Alive without LT |
| 13 | 38 | F | CTD-ILD | Consistent | SLB | - | Unclassfiable |  | Alive without LT |
| 14 | 59 | M | IPF | Consistent | SLB | - | UIP |  | Deceased before LT |
| 15 | 37 | M | Other IIPs | Consistent | SLB | - | Unclassfiable |  | Alive without LT |
| 16 | 50 | F | Other IIPs | Consistent | SLB | - | UIP |  | Deceased before LT |

Abbreviations: PPFE, plueroparenchymal fibroelastosis; F, female; M, male; LONIPC, late-onset non-infectious pulmonary complication after hematopoetic stem-cell transplantation and/or chemotherapy; IPF, idiopathic pulmonary fibrosis; other IIPs, other idiopathic interstitial pneumonias than idiopathic pulmonary fibrosis; SLB, surgical lung biopsy; BO, bronchiolitis obliterans; NSIP, non-specific interstitial pneumonia; LT, lung transplantation
